# Tachycardia-induced unexpected pacemaker behaviour

**DOI:** 10.1007/s12471-014-0606-0

**Published:** 2014-10-22

**Authors:** A. Bőhm, R. G Kiss, G. Z. Duray

**Affiliations:** Department of Cardiology, Military Hospital, Róbert K körut 44, Budapest, Hungary 1344

At the beginning of the recording, atrial tachycardia (AT) can be observed with 1:1 conduction at ventricular rate 140 bpm (upper track). At the spontaneous termination of the tachycardia, atrial-ventricular pacing at 45 bpm (lower rate) and after two cycles atrial-ventricular pacing at 100 beats/min (upper track) can be observed.

**Fig. 1:**
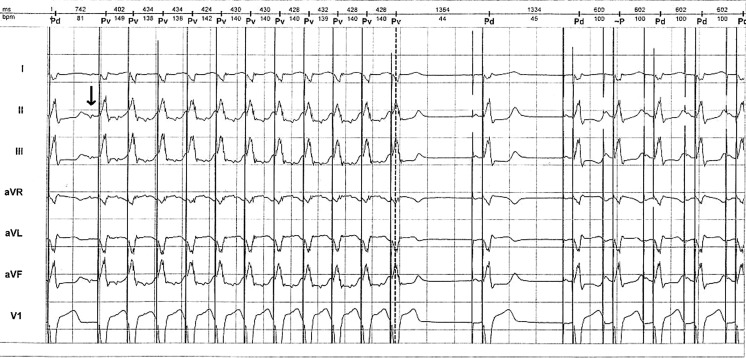
Holter recording of rate drop response induced by paroxysmal atrial tachycardia

The rate drop response was activated: drop size 25 ppm, drop rate 50 ppm, detection beat 2, detection window 1 min, intervention rate 100 ppm, intervention duration 2 min (Fig. 1).

The termination of atrial tachycardia is followed by 45 bpm pacing, which is lower than the drop rate (50 bpm) while the rate drop is higher than the drop size (25 bpm). These two parameters can activate rate drop response separately and in combination, resulting in 100 bpm pacing.

The upper track was reduced to 100 bpm and AT ablation was suggested.

